# Lipoprotein levels and cardiovascular risk in HIV-infected and uninfected Rwandan women

**DOI:** 10.1186/1742-6405-7-34

**Published:** 2010-08-26

**Authors:** Kathryn Anastos, François Ndamage, Dalian Lu, Mardge H Cohen, Qiuhu Shi, Jason Lazar, Venerand Bigirimana, Eugene Mutimura

**Affiliations:** 1 Montefiore Medical Center, Bronx NY, USA; 2 Albert Einstein College of Medicine, Bronx, NY USA; 3 TRAC Plus - Center for Treatment and Research on AIDS, Malaria, Tuberculosis and other Epidemics, Kigali, Rwanda; 4 Data Solutions LLC, Bronx, NY USA; 5 Stroger (Cook County) Hospital and Rush University, Chicago, Illinois USA; 6 New York Medical College, Valhalla, NY USA; 7 SUNY Downstate Medical Center, Division of Cardiovascular Medicine, Brooklyn NY USA; 8 King Faisal Hospital, Kigali, Rwanda; 9 Women's Equity in Access to Care and Treatment (WE-ACTx) and Kigali Health Institute, Kigali, Rwanda

## Abstract

**Background:**

Lipoprotein profiles in HIV-infected African women have not been well described. We assessed associations of lipoprotein levels and cardiovascular risk with HIV-infection and CD4 count in Rwandan women.

**Methods:**

Cross-sectional study of 824 (218 HIV-negative, 606 HIV+) Rwandan women. Body composition by body impedance analysis, CD4 count, and fasting serum total cholesterol (total-C), triglycerides (TG) and high-density lipoprotein (HDL) levels were measured. Low-density lipoprotein (LDL) was calculated from Friedewald equation if TG < 400 and measured directly if TG ≥ 400 mg/dl.

**Results:**

BMI was similar in HIV+ and -negative women, < 1% were diabetic, and HIV+ women were younger. In multivariate models LDL was not associated with HIV-serostatus. HDL was lower in HIV+ women (44 vs. 54 mg/dL, p < 0.0001) with no significant difference by CD4 count (p = 0.13). HIV serostatus (p = 0.005) and among HIV+ women lower CD4 count (p = 0.04) were associated with higher TG. BMI was independently associated with higher LDL (p = 0.01), and higher total body fat was strongly associated with higher total-C and LDL. Framingham risk scores were < 2% in both groups.

**Conclusions:**

In this cohort of non-obese African women HDL and TG, but not LDL, were adversely associated with HIV infection. As HDL is a strong predictor of cardiovascular (CV) events in women, this HIV-associated difference may confer increased risk for CV disease in HIV-infected women.

## Introduction

Dyslipidemias have been described since 1989 in individuals with human immunodeficiency virus (HIV) infection in resource-replete countries, prior to the availability of combination antiretroviral therapy (cART), in studies primarily of white men [1-6]. Quantitative abnormalities include higher triglyceride levels (TG) [2-6] and lower levels of total cholesterol (total-C) [1,5-7], high-density lipoprotein (HDL) [3,6,8] and low-density lipoprotein (LDL) [1-7,9] cholesterol. These abnormalities are greater with more advanced immune suppression [2-6]. Patterns of lipoprotein changes are somewhat different in HIV-infected women [10], with most studies showing no association of HIV-infection with lower LDL. Both women and African Americans in general have higher HDL and lower TG than do European American men [11,12]. Further information is required regarding lipoprotein profiles and potential cardiovascular risk in HIV-infected Africans, who start with lower total-C, TG and LDL, and higher HDL, compared to individuals of European descent living in resource-replete settings. A South African study found lower HDL and LDL and higher TG in HIV-infected compared to uninfected patients [13], and a study in Uganda has reported little change in lipoprotein levels over a two-year period in patients initiating cART [14].

In sub-Saharan Africa and the United States, women and/or people of African descent represent the majority of those infected with and treated for HIV-infection. Because cardiovascular disease (CVD) is a growing concern for HIV infected individuals [15,16] and in developing countries, and dyslipidemia is a major risk factor for CVD, we assessed cardiovascular risk and predictors of lipoprotein levels in HIV-infected and uninfected Rwandan women.

## Methods

### Study Population

The Rwanda Women's Interassociation Study and Assessment (RWISA) is an observational prospective cohort study of HIV-infected and uninfected Rwandan women investigating the effectiveness and toxicity of antiretroviral therapy. In 2005, 710 HIV-infected and 226 HIV-uninfected women were enrolled with follow-up visits occurring every six months. Informed consent was obtained in accordance with protocols approved by the Rwandan National Ethics Committee and the Institutional Review Board of Montefiore Medical Center, Bronx NY, USA. Participants were recruited through grassroots women's organizations and clinical care sites for HIV-infected patients. Inclusion criteria were: age 25 years or older at study entry, willingness to give informed consent, presence in Rwanda during 1994 and no history of receiving antiretroviral treatment except single dose nevirapine to prevent mother-to-child transmission of HIV. At study entry participants provided historical information including socio-demographics, medical and psychosocial history and symptoms, and experience of trauma during the 1994 Rwandan genocide. A physical examination and body impedance analysis (BIA) were performed, and fasting blood specimens were taken. This cross-sectional analysis includes enrollment data from 824 participants for whom fasting lipoprotein levels were measured at study enrollment.

### Laboratory Methods

Total-C, triglyceride and HDL analysis and albumin determination (as a proxy biochemical measure of nutritional status), were performed in the laboratory of King Faisal Hospital in Kigali, Rwanda using standard laboratory methods. For 112 women lipid values were not available because of a lack of reagents at the time of their study visits. LDL was calculated with the Friedewald equation [17], using measured values for total cholesterol, HDL and triglycerides. [LDL = (total-C) - (HDL) - (Trig/5.0)]. If triglyceride level was ≥ 400 mg/dL (n = 11), LDL was measured directly by Quest Laboratory in Baltimore Maryland USA. CD4 counts were determined with a FACS counter (Becton and Dickinson, Immunocytometry Systems, San Jose, CA, USA) at the National Reference Laboratory of Rwanda.

### Exposure variables

The primary exposure variables were HIV serostatus (positive vs. negative) and in the HIV-positive women CD4 cell count, both as a continuous variable (per 100 cells/μl increment) and categorized as < 200, 200-350, and > 350 cells/μl. Secondary exposure variables included age, income, educational attainment, body mass index (BMI) calculated as weight in kilograms divided by (height in meters)^2^, total body fat (TBF) and lean body mass calculated from BIA body composition measures and weight, self-reported menopausal status, current tobacco smoking (yes vs. no), history of diabetes mellitus defined by self-report or fasting plasma glucose > 125 mg/dL, and a reported perception of having adequate food. HIV-1 RNA was included for the 271 HIV+ women whose value was measured.

### Outcome Variables

The primary outcomes of interest were total-C, LDL, HDL, and TG, and calculated Framingham 10-year CVD risk.

### Statistical Methods

Analysis of variance or chi-square tests were used to compare mean lipid levels and percent of participants with abnormal lipid levels among groups defined by HIV status and CD4 count. Variables included in the multivariate models were chosen by their association with lipoprotein values in univariate analysis; variables with p < 0.10 were included in the multivariable models analyzing the outcome variables among the groups. If the overall ANOVA test for differences in lipid levels among > 2 groups was significant, two-sample tests were used to assess pair-wise comparisons. Otherwise, pair-wise comparisons are not reported. In assessment of the Framingham Risk Score (FRS) we performed an age-matched analysis, with each pair randomly selected with the age-difference within +/- 2 years. We also calculated FRS without including the age component. All analyses were performed using the SAS statistical package (Version 8, SAS Institute, Cary, NC).

## Results

Demographic and clinical characteristics of the 606 HIV+ and 218 HIV-negative participants included in this analysis are shown in Table 1. The HIV+ women were younger and were less likely to be post-menopausal. Less than 4% of women in either group used tobacco or illicit drugs, and 0.5% were diabetic.

**Table 1:** Participant Characteristics, Unadjusted Lipoprotein Levels, by HIV Serostatus and CD4 Count

	HIV negative N = 218	HIV+ women (all) N = 606	P-value	HIV+ CD4 > 350 n = 158	HIV+ CD4 200-350 n = 224	HIV+ CD4 < 200 n = 222	P-value
**Age (years) Mean (SD)**	42.4 (10.5)	34.7 (6.8)	< 0.0001	34.0 (6.8)	34.9 (6.5)	34.9 (7.2)	0.35

**Body Mass Index (Kg/m^2^)**							

*Median (IQR)*	20.6 (18.6,23.5)	21.1 (19.1, 23.4)	0.40	21.7 (19.0,23.8)	21.3 (19.2,24.0)	20.9 (19.0,23.1)	0.068

**Total Body Fat (Kg)**							

*Median (IQR)*	11.4 (6.6, 19.0)	12.0 (7.9, 17.0)	0.95	11.4 (7.9, 16.5)	12.1 (7.75, 17.5)	12.1 (7.9, 16.4)	0.43

**Lean Body Mass (Kg)**							

*Median (IQR)*	41.8 (38.7, 44.9)	40.0 (37.2, 43.0)	0.0006	40.5 (38.4, 43.5)	40.4 (37.5, 44.2)	39.3 (35.9, 42.0)	0.013

**Post-menopausal (%)**	22.2	5.7	< 0.0001	6.6	4.9	5.9	0.79

**# Sexual partners^1 ^**							

*Mean (SD)*	3.6 (5.4)	7.5 (22.7)	0.015	6.3 (15.0)	7.5 (23.9)	8.5(26.0)	0.66

*Median (IQR)*	2 (1, 4)	3 (2, 6)		3 (2, 5)	3 (2, 5)	3 (2, 6)	

**Smoking (%)**	3.4	2.5	0.50	2.6	2.7	2.3	0.96

**Alcohol**			0.40				0.73

*None*	77.1	81.0		83.5	80.4	79.3	

*1-3 drinks/day*	22.5	18.3		16.5	18.8	19.4	

* > 3 drinks/day*	0.5	0.7		0.0	0.9	0.9	

**Illicit drug use (%)**	3.5	2.7	0.57	1.9	2.7	3.2	0.74

**Food adequacy (%)**			0.41				0.67

*Always*	0.5	1.6		2.0	0.9	2.1	

*Most of the time*	6.7	5.4		6.8	4.2	5.6	

*Some of the time*	48.3	43.2		41.9	48.1	39.2	

*Usually not*	32.3	37.5		35.1	36.1	40.2	

*Never*	12.1	12.3		14.2	10.7	12.9	

**Running water in home (%)**	4.1	4.8	0.70	5.9	4.5	4.3	0.76

**Education (%)**			0.0002				

*No schooling*	36.2	23.3		24.7	21.0	24.8	0.32

*Grade 1-6*	52.8	67.0		68.4	70.1	62.6	

*Grade 7-11*	7.8	8.4		7.0	7.1	10.8	

*Completed high school*	2.3	1.3		0.0	1.8	1.8	

*Some college*	0.9	0.0		0.0	0.0	0.0	

**Monthly Income $US** *Mean (SD)*	38 (37.0)	38 (30.3)	0.98	38 (30.2)	37 (30.3)	39 (30.4)	0.89

**Diabetes Mellitus (%)**	0.5	0.5	0.98	0.7	0.5	0.5	0.96

**CD4 cell count (cells/mm^3^)**							

*Mean (SD)*	878 (299)	275 (163)	< 0.0001	487 (137)	274 (43)	124 (51)	< 0.0001

HIV-1 RNA* (log_10_)		n = 271		n = 34	n = 93	n = 143	

*Mean (SD)*	---	4.06 (1.37)	---	3.73 (1.40)	3.94 (1.39)	4.22 (1.35)	0.100

**Lipoprotein Levels, mg/dl**							
*Mean (SD)*							

*LDL*	71 (29.7)	68 (28.9)	0.20	67 (25.3)	68 (26.)	67 (33.3)	0.98

*HDL*	54 (16.9)	44 (17.3)	< 0.0001	46 (14.5)	45 (19.5)	43 (16.7)	0.13

*Non-HDL*	89 (33.6)	86 (31.2)	0.21	84 (27.6)	86 (30.7)	87 (34.3)	0.59

*Triglycerides*	87 (42.0)	94 (47,9)	0.11	82 (32.6)	93(51.6)	102 (50.9)	0.003

*Total Cholesterol*	143 (36.4)	129 (33.9)	< 0.0001	129 (31.5)	130 (32.4)	130 (37.1)	0.92

^1 ^includes rape; *HIV-1 RNA levels available for 271 women; LDL = low density lipoprotein cholesterol; HDL = high density lipoprotein cholesterol, Non-HDL = Total Cholesterol - HDL.

### Associations with HIV serostatus and CD4 cell count

Unadjusted and adjusted mean lipoprotein values are shown in Tables 1 and 2 respectively. In both adjusted and unadjusted analyses, mean LDL and non-HDL were similar in the HIV-positive and negative women and did not vary by CD4 count in the HIV+ participants. In adjusted models (Table 2), total-C (p = 0.014) and HDL (p < 0.0001) were lower in HIV+ than in HIV-negative participants without significant variation by CD4 count. The differences in total-C were driven predominately by the differences in the HDL component. In multivariate analysis TG in the HIV+ women was significantly higher at lower CD4 counts (p = 0.040), and in HIV+ compared to HIV-negative women (p = 0.005).

**Table 2:** Multivariate associations of Lipoprotein levels with HIV serostatus and CD4 lymphocyte count in 824 participants

**Lipoprotein Levels*****Mean (Standard error)***	HIV-negative N = 218	HIV-positive (all) N = 606	P-value	HIV+ CD4 > 350 N = 158	HIV+ CD4 200-350 N = 224	HIV+ CD4 < 200 N = 222	P-value
*LDL^1^*	69 (3.0)	66 (1.7)	0.363	68 (3.2)	65 (2.8)	66 (2.7)	0.74

*HDL^2^*	54 (1.3)	44 (0.8)	< 0.0001	46 (1.4)	42 (1.2)	43 (1.3)	0.097

*Non-HDL^3^*	85 (2.5)	87 (1.5)	0.549	85 (2.7)	87 (2.3)	85 (2.4)	0.74

*Triglycerides^4^*	79 (5.0)	96 (2.9)	0.005	82(5.7)	93 (5.0)	101(4.7)	0.040

*Total Cholesterol^5^*	139 (2.8)	130 (1.7)	0.014	131 (3.0)	130 (2.6)	128 (2.7)	0.73

*All models adjusted for age, menopausal status, BMI, income, education, alcohol use, smoking and food availability.

LDL = low density lipoprotein cholesterol; HDL = high density lipoprotein cholesterol, Non-HDL = Total Cholesterol - HDL.

### Associations of lipid levels with demographic and clinical variables and measures of nutritional status

In multivariate analyses including HIV serostatus, age, BMI, menopause, smoking, alcohol use, income, education and perception of food availability only BMI was independently associated with higher LDL (p = 0.01). Higher HDL was associated with increased alcohol use (p < 0.001). Age was independently associated with TG (p = 0.005), and higher non-HDL was associated with post-menopausal status (p = 0.010), smoking (p = 0.025) and BMI (p = 0.0001). In additional multivariate analysis that included albumin, total body fat and lean body mass, and adjusting for demographic variables, all measures of nutritional status were strongly associated with each of the lipoprotein levels. A 1.0 mg/dL higher serum albumin was associated with large significant increases in total-C (18 mg/dL), LDL (7 mg/dL) and HDL (10 mg/dL, p < 0.0001 for all, data not shown). Higher total body fat (Table 3) was strongly associated with higher total-C and LDL (p < 0.0001 and = 0.001, respectively). Similar patterns in the associations of TBF with lipoprotein levels were seen in the HIV-negative women but were not statistically significant (data not shown).

**Table 3:** Quartiles of total body fat adjusted for CD4 lymphocyte count in HIV-positive women

Lipoprotein Levels ***Mean (Standard Deviation)***	Total Body Fat in Kilograms				P-value
	**≤ 7.4**	**7.4 to 11.8**	**11.8 to 17.4**	**> 17.4**	

*N*	113	135	135	118	

*LDL*	58.5 (28.1)	66.1 (28.2)	66.3 (27.3)	77.7 (30.1)	0.001

*HDL*	42.7 (16.5)	42.6 (16.6)	43.0 (15.2)	44.7 (12.4)	0.69

*Non-HDL*	78.5 (29.2)	81.6 (26.9)	87.3 (26.8)	98.8 (36.2)	< 0.0001

*Triglycerides*	98.6 (46.4)	90.6 (39.2)	97.6 (61.0)	94.2 (38.7)	0.62

*Total Cholesterol*	120.6 (30.7)	124.1 (31.3)	130.2 (32.0)	141.8 (39.8)	< 0.0001

LDL = low density lipoprotein cholesterol; HDL = high density lipoprotein cholesterol, Non-HDL = Total Cholesterol - HDL.

### Framingham Risk Scores

Framingham 10 year cardiovascular risk (Table 4) was extremely low in all participants with little variation by HIV serostatus: 2.04% vs. 1.22% in HIV-negative and HIV+ women respectively (p < 0.0001). Because of the older age of the HIV-negative women, we conducted two additional analyses, and found no significant difference between HIV-negative and HIV+ women in age-matched analysis of 154 HIV-negative and 154 HIV+ women (1.57 and 1.63 respectively, p = 0.72) or when risk scores were calculated without the age component (1.36 and 1.22, p = 0.15).

**Table 4:** Framingham 10-year risk scores by HIV-serostatus

	HIV negative N = 218	HIV-positive N = 606	P-value
**Framingham 10-year risk (FRS)**	2.04 (1.7)	1.22 (1.0)	< 0.0001

*FRS without age component*	1.36 (1.2)	1.22 (1.0)	0.15

*FRS age-matched (n = 308)*	1.57 (1.2)	1.63 (1.6)	0.72

## Discussion

In this study of HIV-negative and ART-naïve HIV+ Rwandan women we found associations of serum lipoprotein levels with HIV serostatus and CD4 count that were similar to those described in United States women [10]. HDL was markedly lower in the HIV+ women with some variation by CD4 count, with values very similar to those in US women, and TG levels were higher with HIV+ serostatus and advanced immune suppression. As in US women, but unlike US men, LDL levels did not differ significantly by HIV serostatus or CD4 count [9,10]. The weak association of lower total-C in HIV+ women was entirely explained by the lower HDL. Unlike HDL, in our study LDL, TG and total-C were not similar to values in US women--all were markedly lower.

The clinical importance of abnormal lipoprotein levels lies primarily in the greater risk they confer for development of CVD [18,19], and there is growing concern about premature CVD in both untreated and cART-treated HIV+ persons [15,16]. However, the lipoprotein profiles of the HIV-negative RWISA participants are quite favorable by United States standards, and the changes associated with HIV-infection may thus not be clinically significant. Still, subclinical atherosclerosis has been found to be more common in HIV+ men independent of FRS [20] and higher HDL is a powerful protective factor for cardiovascular disease in women, of greater importance than it is in men [21,22]. The very low HDL in untreated HIV+ women and the moderately higher TG levels may indeed increase CVD risk in African women, and further information is needed to determine whether clinical CVD outcomes will increase with HIV treatment. Only 15% of HIV-negative and 11% of HIV+ women had LDL levels above 100 mg/dL, the most stringent target when treating LDL levels in the United States.

Some cART regimens are associated with worse lipoprotein profiles, especially protease inhibitors (PI) [9,23,24] and stavudine [25,26]. The non-nucleoside reverse transcriptase inhibitors (NNRTI) have been associated with beneficial effects on HDL [24,25]. The NNRTIs are the most common anchor drugs in cART regimens in Africa, and stavudine is increasingly avoided. Thus African women initiating ART may benefit with improved lipoprotein profiles, especially higher HDL. Follow-up of Africans initiating cART will improve our understanding of its effects on lipoprotein levels, potential CVD risk, and the actual rates of CVD events.

We found that the total body fat did not impact meaningfully on the HDL and TG values associated with HIV disease. However, TBF was significantly associated with higher LDL, non-HDL and total cholesterol. The trends in associations of lipid levels with TBF were similar in the HIV+ and negative women. Thus body composition was not informative in assessing the impact of HIV immune suppression.

Limitations of our study include its cross-sectional design and the absence of information on diet and exercise. Strengths include the relatively large sample size, the uniformity of specimen and data collection, and the inclusion of body composition measures.

## Conclusion

We found that lower HDL and higher TG were the primary adverse lipoprotein levels associated with HIV infection in Rwandan women, while LDL was less affected. Because HDL is a strong predictor of CVD outcomes in women, this HIV-associated difference may carry significant risk of CVD in HIV-infected women. Longitudinal studies of African women receiving cART are needed to define these patterns in the presence of HIV treatment and to ascertain the associated cardiovascular risk.

## Competing interests

The authors declare that they have no competing interests.

## Authors' contributions

KA obtained funding, designed and implemented the study, participated in data analysis, and wrote the manuscript; FN assisted in writing early drafts; DL performed data analysis; MHC helped with study implementation and reviewed and commented on manuscript; VB performed laboratory analyses, and reviewed and commented on manuscript; JL provided technical expertise and reviewed and revised manuscript; EM participated in manuscript writing and preparation. All authors have read and approved the final manuscript.
